# Differential expression patterns of phospholipase D isoforms 1 and 2 in the mammalian brain and retina

**DOI:** 10.1016/j.jlr.2022.100247

**Published:** 2022-06-25

**Authors:** Casey N. Barber, Hana L. Goldschmidt, Brendan Lilley, Alexei M. Bygrave, Richard C. Johnson, Richard L. Huganir, Donald J. Zack, Daniel M. Raben

**Affiliations:** 1 Department of Biological Chemistry, The Johns Hopkins University School of Medicine, Baltimore, MD, USA; 2 Solomon H. Snyder Department of Neuroscience, The Johns Hopkins University School of Medicine, Baltimore, MD, USA; 3 Department of Ophthalmology, Wilmer Eye Institute, The Johns Hopkins University School of Medicine, Baltimore, MD, USA

**Keywords:** phospholipids/phosphatidic acid, brain lipids, eye/retina, phospholipases/D, exocytosis, neurotransmission, synaptic plexiform layer, glia, CRISPR/Cas9, knock-in mice, BSA, bovine serum albumin, DIV, days in vitro, ERG, electroretinogram, GFAP, glial fibrillary acidic protein, NGS, normal goat serum, PBST, phosphate-buffered saline containing 0.1% Triton-X, PKC, protein kinase C, PLD, phospholipase D, PtdOH, phosphatidic acid, vGlut1, vesicular glutamate transporter 1

## Abstract

Phosphatidic acid is a key signaling molecule heavily implicated in exocytosis due to its protein-binding partners and propensity to induce negative membrane curvature. One phosphatidic acid-producing enzyme, phospholipase D (PLD), has also been implicated in neurotransmission. Unfortunately, due to the unreliability of reagents, there has been confusion in the literature regarding the expression of PLD isoforms in the mammalian brain which has hampered our understanding of their functional roles in neurons. To address this, we generated epitope-tagged PLD1 and PLD2 knockin mice using CRISPR/Cas9. Using these mice, we show that PLD1 and PLD2 are both localized at synapses by adulthood, with PLD2 expression being considerably higher in glial cells and PLD1 expression predominating in neurons. Interestingly, we observed that only PLD1 is expressed in the mouse retina, where it is found in the synaptic plexiform layers. These data provide critical information regarding the localization and potential role of PLDs in the central nervous system.

Phosphatidic acid (PtdOH) is a multi-functional lipid second messenger heavily implicated in membrane fusion in many cell types including adipocytes, macrophages, and neuroendocrine cells (1, 2). Although PtdOH is typically only present in limited amounts with a high turnover rate in mammalian cells, it is a key metabolite involved in many signaling events and biosynthetic pathways, perhaps most notably in membrane fusion of secretory vesicles and granules (3, 4, 5). PtdOH is negatively charged with a small headgroup, making it likely to a) assemble into microdomains through intermolecular hydrogen bonding that serve as membrane insertion sites, b) promote membrane curvature, and c) induce conformational changes of proteins. For example, PtdOH activates phosphatidylinositol kinase and binds key fusion proteins such as syntaxin-1 (6, 7). Furthermore, it is the metabolic intermediate for other signaling lipids such as diacylglycerol (DAG) and lyso-PtdOH (8). Despite the accumulating evidence for the involvement of PtdOH in exocytosis (2, 6, 7, 9, 10), its role in this process has not been completely elucidated in neurons.

De novo synthesis of PtdOH can occur through acylation of glycerol 3-phosphate or dihydroxyl acetone phosphate. It may also be metabolically generated by three pathways: 1) diacylglycerol kinase-mediated phosphorylation of diacylglycerol, 2) acylation of lyso-PtdOH by lyso-PA-acyltransferase, and 3) phospholipase D (PLD)-mediated hydrolysis of phospholipids such as phosphatidylcholine (9). While there is a growing body of work supporting the hypothesis that PLD-generated PtdOH is critical for exocytosis in neuroendocrine cells, PLD remains virtually uncharacterized in synaptic vesicle recycling in neurons.

PLD has 6 isoforms, although only PLD1 and PLD2 reportedly have canonical PLD enzymatic activity (11). PLD1 and PLD2 both contain two HxK(x)_4_D (H-histidine, K-lysine, D-aspartic acid) domains that together confer the enzyme’s catalytic activity. Any point mutation within these regions results in a decrease or absence of PLD enzymatic activity (10). Additionally, they both contain a phox homology domain, known to mediate protein-protein interactions; a plekstrin homology domain that is not required for enzymatic activity but regulates localization; and a phosphatidylinositol 4,5-biphosphate-binding site, which is required for catalytic activity. PLD1 also contains a loop sequence that is absent in PLD2 (12, 13). While PLD2 has a high basal activity alone, PLD1 requires activation by ADP-ribosylation factor, Rho and Ral GTPases, or protein kinase C (PKC) (14, 15). Research over the last 30 years suggests a role for PLD in cell proliferation and differentiation (16, 17, 18) but also in Ca^2+^-regulated exocytosis. Importantly, Humeau *et al.* demonstrated that the injection of catalytically inactive PLD1 into *Aplysia* cholinergic neurons resulted in a rapid, dose-dependent inhibition of acetylcholine release. This, together with the wealth of evidence implicating PtdOH in exocytosis, led us to hypothesize that a PLD may have a regulatory role in neurotransmission at mammalian cortical synapses or in ribbon synapses of the retina.

Unfortunately, due to conflicting results and ambiguity in the existing literature (19, 20, 21, 22), it is difficult to draw concrete conclusions about the expression and localization of PLD1 and PLD2 in the mammalian brain. While there are a number of reasons for this, the reagents used in these studies may be problematic (23). For example, three studies using the same commercially-available PLD1 antibody reported conflicting expression profiles of PLD1 in cortical and hippocampal neuron cultures (24, 25, 26). There are also conflicting reports about the presence of PLD isoforms in glial cells (23, 24).

To address this controversy regarding the expression and localization of PLD1 and PLD2 in the mammalian brain, we generated endogenously expressed, epitope-tagged PLD1 and PLD2 knockin mice. Using neuron cultures and brain slices from these mice, we were able to demonstrate the expression of both PLD1 and PLD2 in mature neurons, with PLD2 expression being much higher in glial cells throughout development and into maturity. Additionally, we observed the presence of PLD1 exclusively in the mouse retina in the synaptic plexiform layers. Taken together, this is the first study to use PLD knockin mice to examine the expression of PLD1 and PLD2 in neuronal and glial cultures, as well as in the brain and retina. Our results clearly demonstrate that PLDs are differentially expressed throughout the central nervous system.

## Materials and methods

### Reagents

**Table:** 

**Primary Antibodies**	**Source**	**Product Number**	**IF Dilution**	**WB Dilution**
Mouse anti-HA ascites	Homemade	N/A	1:1000	1:2500
Mouse anti-myc ascites	Homemade	N/A	1:1000	1:2000
Guinea pig anti-vGlut1	Synaptic Systems	135304	1:2500	-
Rabbit anti-GFAP	Cell Signaling Technology	12389S	1:200	-
Rabbit anti-PKCα	Cell Signaling Technology	59754S	1:100	-
**Secondary Antibodies**	**Source**	**Product Number**	**IF Dilution**	**WB Dilution**
IRDye 680LT goat anti-mouse IgG	LICOR	926–68020	-	1:10,000
Goat anti-Guinea Pig Alexa Fluor 647	Invitrogen	A21450	1:500	-
Goat anti-Mouse Alexa Fluor 647	Invitrogen	A21236	1:500	-
Goat anti-Rabbit Alexa Fluor 568	Invitrogen	A11036	1:500	-

### Knockin mice

All animal protocols were approved by the Johns Hopkins University Animal Care and Use Committee. To generate myc-PLD1 knockin and HA-PLD2 knockin mouse lines, CRISPR-Cas9 was used to insert tags into the N-terminal region of the protein sequence. Three tandem myc tags were inserted into exon 2 (the first protein-coding exon) of PLD1, and three tandem HA tags were inserted into exon 2 (the first protein-coding exon) of PLD2. Both insertion points were upstream of the phox homology domain within each protein. Pronuclear injection of one-cell C57BL/6J embryos (Jackson Laboratories, 000664) was performed by the JHU Transgenic Core using standard microinjection techniques (27) using a mix of Cas9 protein (30 ng/μl, PNABio), tracrRNA (0.6 μM, IDT), PLD1 crRNA (0.3 μM, IDT), PLD2 crRNA (0.3 μM, IDT), PLD1 ssDNA oligo (5 ng/μl, IDT), and PLD2 ssDNA oligo (5 ng/μl, IDT) diluted in RNAse-free injection buffer (10 mM Tris-HCl, pH 7.4, 0.25 mM EDTA). See table below for sequences. Injected embryos were transferred into the oviducts of pseudopregnant ICR females (Envigo, 030) using previously described techniques (27). Resulting mice were then backcrossed to WT C57BL/6J mice and heterozygotes were crossed to produce mice homozygous for the inserted tags.

**Table:** 

PLD1 crRNA	GUCACUGAAAAGCGAGACCAGUUUUAGAGCUAUGCU
PLD2 crRNA	GAUAGUCCCCAUAGGGAAAGGUUUUAGAGCUAUGCU
PLD1 ssDNA oligo	CCCCGTCAGAAGCTAGCATGTCACTGAAAAGCGAGCAAAAGCTCATTTCTGAGGAAGATCTCGAACAGAAATTGATCAGCGAGGAGGACTTGGAACAGAAGTTGATCAGTGAAGAGGATCTGAGCCTCAAGTCTGAAACTCGCGTGAACACGTCTACACTGCAGAAAATCGCCGCAGA
PLD2 ssDNA oligo	CTCATATTCCTAGGATGACTGTAACCCAGAAGAACTACCCATACGATGTTCCAGATTACGCTGAATTCTATCCTTATGACGTCCCTGACTATGCATATCCTTATGACGTCCCTGACTATGCCACCGTGACACAAAAAAATCTTTTTCCCTATGGGGACTATCTGAACTCCAGCCAGTT

### Harvesting brain tissue

To confirm the presence of myc and HA tags in our PLD1 and PLD2 knockin mouse lines, respectively, WT, myc-PLD1, and HA-PLD2 mice were sacrificed at postnatal day 21 (P21) and brains were removed. Whole brain tissue was homogenized with a glass homogenizer in homogenization buffer (0.32 M sucrose, 4 mM Hepes, pH 7.4) until solution was uniform. The homogenate was transferred to Eppendorf tubes and centrifuged at 1000 *g* for 10 min at 4°C. The supernatant (S1) was collected and transferred to a new tube and kept at −20°C until further analysis.

### Western blotting analysis

SDS-PAGE was performed with Bolt 8% Bis-Tris mini protein gels. S1 from whole brain lysates were diluted with water and 4x Laemmli sample buffer containing 10% β-mercapthoethanol and boiled for 5 min at 100°C before loading on gels. Following electrophoresis, proteins were transferred to nitrocellulose membranes with the Bio-Rad Mini Trans-Blot system and blocked with 3% bovine serum albumin (BSA) for 1 h. Blots were incubated with primary antibodies diluted in 3% BSA in phosphate-buffered saline containing 0.1% Triton-X (PBST) on a 4°C rocker overnight. The next day, blots were washed 3× with PBST and incubated with LICOR IRDye-conjugated secondary antibodies for 1 h at room temperature. Blots were then washed 3× with PBST before infrared imaging using an Odyssey Imaging System.

### Neuron culture

To prepare mouse primary neuron cultures, brains were removed from P0 pups. Cortices were incubated in dissection media with papain and DNase for 25 min at 37°C and then fully dissociated with gentle trituration. Cells were then plated on poly-L-lysine-coated 18 mm glass coverslips placed in the wells of a 12-well plate in Neurobasal Plus medium supplemented with 5% horse serum, 50 U/ml penicillin, 50 U/ml streptomycin, 2 mM Glutamax, and 2% b27 Plus at 500k cells/well. Neurons were maintained in a 37°C incubator (5% CO_2_). At days in vitro (DIV)4, neurons were fed with serum-reduced (2% horse serum) media supplemented with fluoro-deoxyuridine to prevent the proliferation of glial cells. Subsequently, neurons were fed every 4 days with Neurobasal Plus medium supplemented with 50 U/ml penicillin, 50 U/ml streptomycin, 2 mM Glutamax, and 2% b27 Plus.

### Culture immunofluorescence

To perform immunofluorescence experiments on mouse neurons plated on glass coverslips as described above, the coverslips were first washed 1× with PBS and then incubated in parafix (4% paraformaldehyde (PFA), 4% sucrose in PBS) for 15 min at room temperature. Cells were washed 3× with PBS and then permeabilized for 10 min with 0.25% Triton-X in PBS. They were then washed 3× with PBS and incubated with 10% BSA at 37°C for 1 h to reduce nonspecific antibody binding. Coverslips were then incubated with primary antibodies diluted in 3% BSA overnight at 4°C. The following day, coverslips were washed 3× with PBS and incubated with secondary antibodies (goat conjugated Alexa Fluor 647, 568, or 488) diluted in 3% BSA in PBST for 1 h at room temperature. They were then washed 3× with PBS and mounted on glass slides with Fluoromount-G and stored at 4°C. Images were obtained with an LSM 880-laser scanning confocal microscope (Zeiss) and images were analyzed with ImageJ and Fiji.

### Brain slice immunofluorescence

WT (P25), myc-PLD1 (P26), and HA-PLD2 (P32) mice were deeply anaesthetized and transcardially perfused with PBS followed by 4% PFA, each ice cold. Brains were postfixed in 4% PFA for 4 h at 4°C and then washed 3× with PBS. Brains were kept at 4°C in PBS until slicing. Brains were sliced into 60 μm sections on a vibratome (VT-1000, Leica) and kept in PBS until staining. For staining, slices were washed 3× with PBS before permeabilizing with 0.3% Triton-X in PBST for 20 min at room temperature. Slices were then incubated with 5% normal goat serum (NGS), 0.15% Triton-X in PBST for 1 h at room temperature to reduce background staining. Primary antibodies were diluted in 5% NGS, 0.15% Triton-X in PBST and incubated with slices overnight on a rocker at 4°C. The next day, slices were washed 5× with PBS and incubated with secondary antibodies diluted in 5% NGS, 0.15% Triton-X in PBST overnight on a rocker at 4°C. Finally, slices were washed 5× with PBS and mounted on glass slides with Epredia Lab Vision PermaFluor aqueous mounting medium. Images were obtained with an LSM 880-laser scanning confocal microscope (Zeiss) and images were analyzed with ImageJ and Fiji.

### Retinal slice immunofluorescence

Mouse retinal slices from WT, myc-PLD1, and HA-PLD2 mice were prepared according to established protocols (28). Briefly, mice were anesthetized using isoflurane and decapitated. Both eyes were explanted and incubated in 4% PFA for 30 min. Next, the cornea and lens were removed and eye cups were incubated in 4% PFA for 2 h on ice. Eye cups were washed carefully with 1× PBS and then soaked in 15% sucrose (w/v) in 1× PBS for 3 h, then incubated in 30% sucrose (w/v) in 1× PBS overnight. Tissue was embedded in optimal cutting temperature compound (TIssueTek) and frozen on dry ice. Slices of 12–14 μm thickness were sectioned using a Leica CM3050S cryostat and were subsequently mounted on Superfrost Plus slides (Fisher Scientific).

To perform retinal staining experiments, slides containing WT, myc-PLD1, and HA-PLD2 retinal slices were first washed in 1× PBS three times for 5 min each. Tissue was permeabilized by incubation with 0.25% Triton-X in 1× PBS for 5 min at room temperature. To limit nonspecific staining, slides were incubated in 0.1% Triton-X in 1× PBS + 5% NGS for 1 h at room temperature. Subsequently, slides were incubated with myc/HA, vesicular glutamate transporter 1 (vGlut1), and PKCα primary antibodies diluted in 0.1% Triton-X in 1× PBS + 5% NGS overnight at 4°C. The next day, slides were washed 3× 15 min each with 1× PBS. Slides were then incubated with secondary antibodies (goat conjugated Alexa Fluor 647, 568, or 488) diluted in 0.1% Triton-X in 1× PBS + 5% NGS for 1 h at room temperature in the dark. Slides were then washed 3× 15 min each with 1× PBS and glass slides were placed on top with Epredia Lab Vision PermaFluor aqueous mounting medium. Images were obtained with an LSM 880-laser scanning confocal microscope (Zeiss) and images were analyzed with ImageJ and Fiji.

### Electroretinograms

Electroretinograms (ERGs) were recorded from PLD1 KO and WT mice using the Celeris full-field ERG system from Diagnosys (model D430) with an internal platform heater to maintain body temperature. Mice were dark adapted overnight and anesthetized with 10 mg/kg Ketamine/1 mg/kg xylazine under dim red light conditions. Pupils were dilated using 0.5% tropicamide eye drops (AKORN Inc.), and GenTeal eye gel (0.3% Hypromellose, Alcon) was applied on both eyes before placing the electrodes. ERG responses were obtained using the opposite eye electrode as a reference and an electrode placed into the haunch as a ground electrode. For scotopic measurements, single flash recordings were performed at light intensities of 0.01, 0.1, 1 cd .s/m^2^ of white light with no illumination between flashes. Following 10 min of light adaptation (3 cd .s/m^2^), we obtained photopic flash recordings at light intensities of 3 and 10 cd .s/m^2^ with a background intensity of 0.1 cd/m^2^. Averages of 5 (scotopic) or 10 (photopic) sweeps were computed and analyzed using Espion V6 software from Diagnosys. The a-wave amplitude measured from stimulus onset to the trough of the a-wave and the b-wave amplitude ranging from the trough of the a-wave to the peak of the b-wave. Data was exported to Microsoft Excel, and a- and B-wave values from the left and right eyes were averaged. Data were compiled, plotted, and analyzed in GraphPad Prism.

### PLD assay

PLD activity was assessed with the EnzyChrom Phospholipase D Assay Kit (BioAssay Systems EPPD-100). A standard curve was prepared with four defined amounts of choline diluted in dH_2_0. Known concentrations of WT, myc-PLD1, and HA-PLD2 whole brain lysate were combined with a master mix of assay buffer, purified PLD enzyme, dye reagent, and phosphatidylcholine substrate. Reactions were mixed and incubated for 10 min at 37°C and 570 nm optical density readings were recorded at 10, 15, and 30 min.

## Results

### Validation of knockin mice

**Fig. 1:**
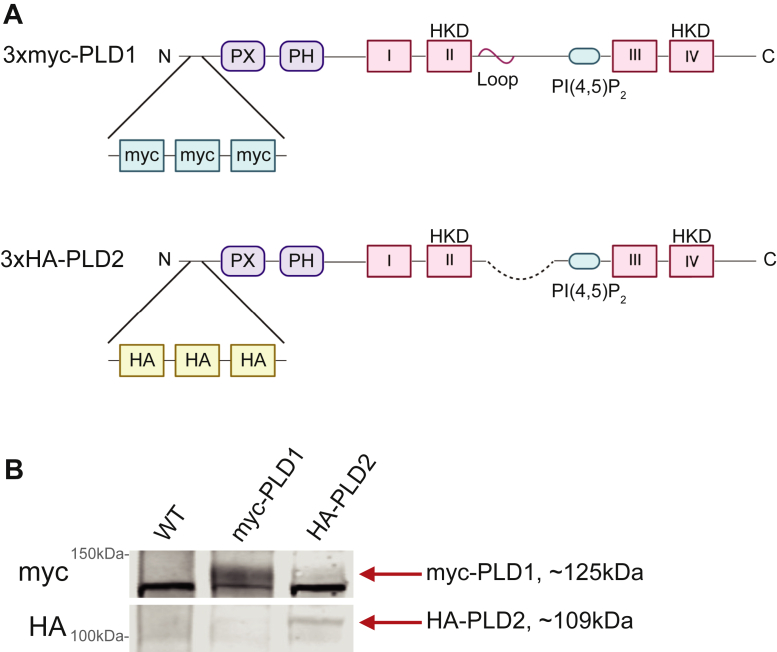
Validation of myc-PLD1 and HA-PLD2 mouse lines. A: Using CRISPR-Cas9, three tandem myc tags were inserted into the N-terminal region of the PLD1 protein, upstream of the phox homology (PX) domain, to generate the myc-PLD1 mouse line. Additionally, three tandem HA tags were inserted into the N-terminal region of the PLD2 protein, upstream of the PX domain. B: Western blots were performed with whole brain lysates from WT, myc-PLD1, and HA-PLD2 mice. A myc antibody visualizes the myc tag in the myc-PLD1 lysate specifically (∼125 kDa), and an HA antibody recognizes the HA band in the HA-PLD2 lysate only (∼109 kDa). PX, phox homology domain; PH, plekstrin homology domain; HKD, HxK(x)_4_D (H-histidine, K-lysine, D-aspartic acid).

To eliminate the need for PLD-specific antibodies for the examination of PLD expression, we generated the first endogenously expressed, epitope-tagged PLD1 and PLD2 knockin mice (see Materials and methods). Using CRISPR-Cas9, three tandem myc tags were inserted into exon 2 of PLD1 and three tandem HA tags were inserted in exon 2 of PLD2 in separate C57BL/6N mouse lines (Fig. 1A). Previous work has shown that even the slightest changes to the C-terminus of PLD proteins will affect enzymatic activity, however, this has not been shown for the N-terminus (29, 30). Mice were genotyped and crossed appropriately to produce homozygous knockins. To confirm the presence of the tags in these mice, adult (P21) brains were collected from WT, myc-PLD1, and HA-PLD2 mice, lysed, and subjected to Western blot analyses. A myc antibody confirmed the presence of the myc tag in the myc-PLD1 brain lysate only, while an HA antibody confirmed the presence of the HA tag in the HA-PLD2 brain lysate only (Fig. 1B). A nonspecific band is observed below the myc-tag band, however, we believe this nonspecificity does not interfere with subsequent experiments due to the lack of myc staining in WT neuron cultures.

To ensure the knockin of myc and HA tags did not interfere with PLD activity in our myc-PLD1 and HA-PLD2 mice, we conducted PLD activity assays with WT, myc-PLD1, and HA-PLD2 whole brain lysates. In this enzyme assay, PLD hydrolyzes phosphatidylcholine to choline, and free choline levels are measured using choline oxidase and a H_2_O_2_-specific dye. With this assay, we determined that there was no significant difference between the amount of choline produced in WT, myc-PLD1, or HA-PLD2 whole brain lysate (supplemental Fig. S1). This data suggests that the addition of myc and HA epitope tags to the N-terminus of PLD1 and PLD2, respectively, does not interfere with PLD activity.

### PLD1 and PLD2 expression in mouse neurons and glia

**Fig. 2:**
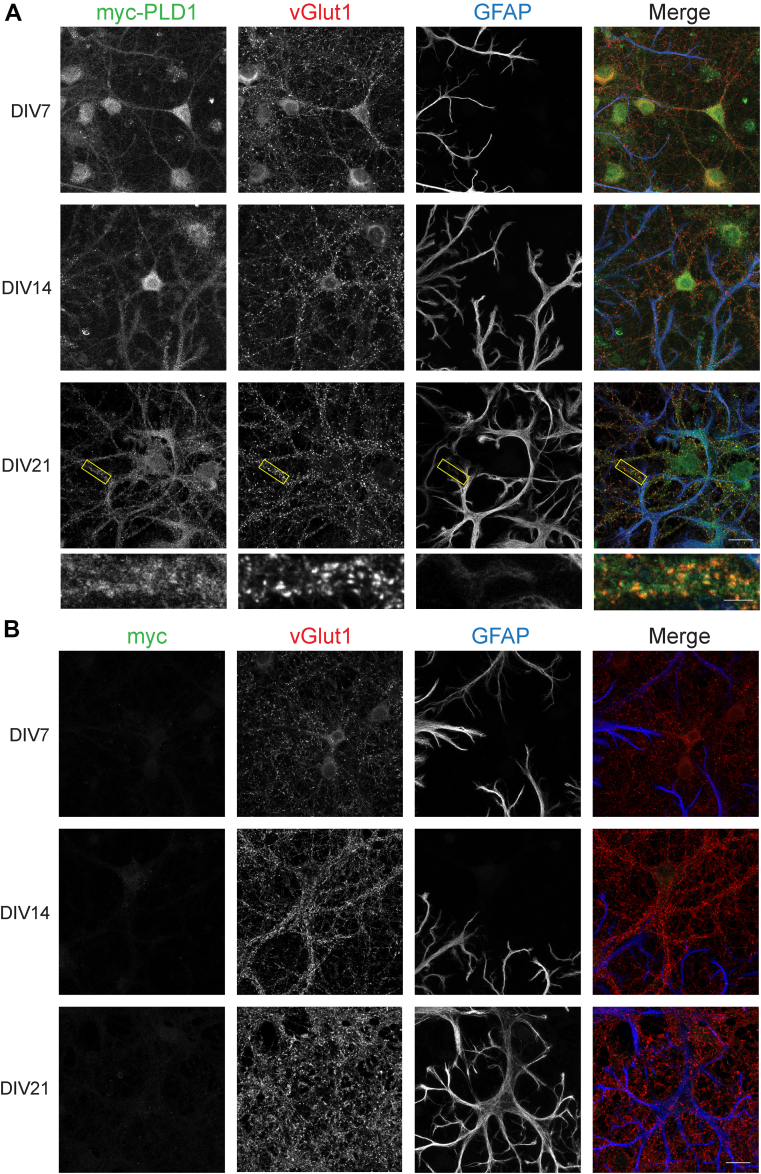
PLD1 is expressed predominantly in neurons, marginally in glia, and is synaptic at DIV21. A: Myc-PLD1 cortical neuron and glia cultures were stained with a myc antibody to visualize myc-PLD1 protein, a vGlut1 antibody as a marker of presynaptic boutons, and a GFAP antibody to visualize glial processes at DIV 7, 14, and 21. B: WT mouse cortical neuron and glia cultures were stained with myc, vGlut1, and GFAP antibodies as in (A). Scale bars represent 20 μm and 5 μm (cropped region). Images are representative of three separate experiments. GFAP, glial fibrillary acidic protein; vGlut1, vesicular glutamate transporter 1.

**Fig. 3:**
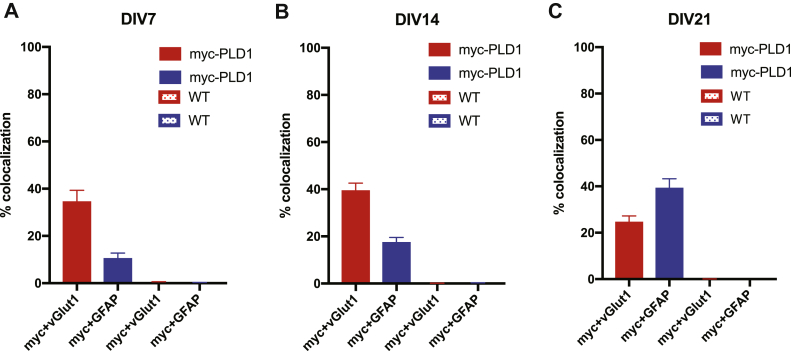
Quantification of myc-PLD1 and WT cortical neuron immunofluorescence. The percent colocalization was calculated for myc + vGlut1 and myc + GFAP in myc-PLD1 and WT cortical neurons at DIV7 (A), DIV14 (B), and DIV21 (C). Data is representative of three experiments and error bars represent SEM. A: myc-PLD1 n = 5, WT n = 3; B, myc-PLD1 n = 6, WT n = 3; C, myc-PLD1 n = 8, WT n = 3. vGlut1, vesicular glutamate transporter 1; GFAP, glial fibrillary acidic protein.

Previous studies on the expression and localization of PLD isoforms in the mammalian brain have resulted in contradictory conclusions. Although PLD1 and PLD2 antibodies are available commercially, we decided to utilize an alternative technique in order to avoid the complexities and potential discrepancies of commercial antibodies against endogenous proteins. This being so, a primary objective of this study was to qualitatively determine whether PLD1 and PLD2 are expressed in neurons or glia throughout development. To address this, we cultured neurons from myc-PLD1 and HA-PLD2 knockin mice and stained for myc/HA, vGlut1, and glial fibrillary acidic protein (GFAP) at DIV 7, 14, and 21. vGlut1 serves as a presynaptic neuronal marker while GFAP is specifically expressed in glial cells (astrocytes). As a control, we also stained WT neuron cultures with the same staining protocols. Interestingly, in myc-PLD1 mouse cortical cultures stained with myc, vGlut1, and GFAP, PLD1 is highly expressed in neurons through development to maturity. At DIV21, myc staining is punctate on dendrites and localizes with vGlut1, suggesting that PLD1 is enriched at synapses. There are marginal increases in PLD1 expression in glial cells throughout development, as evidenced by an increase in colocalization of myc staining with GFAP (Figs. 2A and 3A: myc + vGlut1 34.41 ± 4.92, myc + GFAP 10.38 ± 2.4; Fig. 3B: myc + vGlut1 39.29 ± 3.30, myc + GFAP 17.34 ± 2.23; Fig. 3C: myc + vGlut1 24.49 ± 2.78, myc + GFAP 39.14 ± 4.15). Minimal anti-myc staining is observed in WT cells at any stage of development, indicating that our myc antibody specifically recognizes PLD1 in these immunofluorescence experiments (Figs. 2B and 3A: myc + vGlut1 0.54 ± 0.07, myc + GFAP 0.19 ± 0.16; Fig. 3B: myc + vGlut1 0.08 ± 0.05, myc + GFAP 0.24 ± 0.07; Fig. 3C: myc + vGlut 0.05 ± 0.02, myc + GFAP 0.04 ± 0.02).

**Fig. 4:**
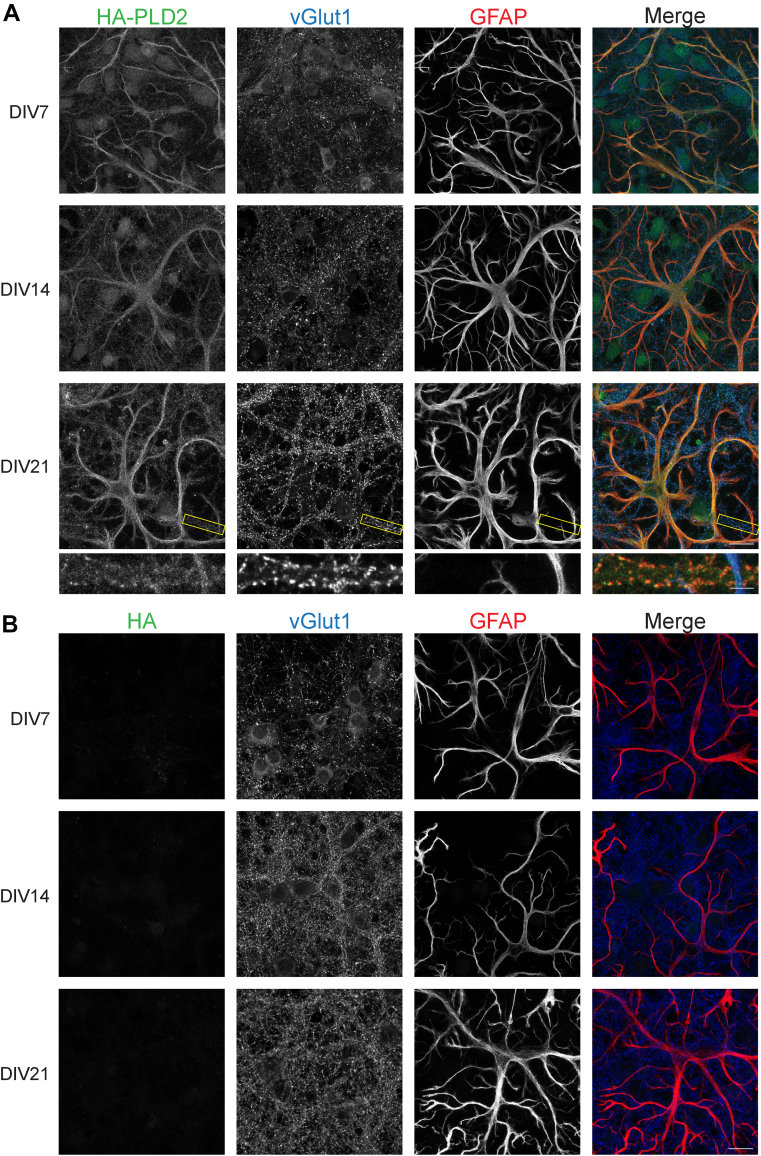
PLD2 expression is robust in glia, moderate in neurons, and is synaptic at DIV21. A: HA-PLD2 cortical neuron and glia cultures were stained with an HA antibody to visualize PLD2 protein, a vGlut1 antibody as a marker of presynaptic boutons, and a GFAP antibody to visualize glial processes at DIV 7, 14, and 21. B: WT mouse cortical neuron and glia cultures were stained with HA, vGlut1, and GFAP antibodies as in (A). Scale bars represent 20 μm and 5 μm (cropped region). Images are representative of three separate experiments. GFAP, glial fibrillary acidic protein; vGlut1, vesicular glutamate transporter 1.

**Fig. 5:**
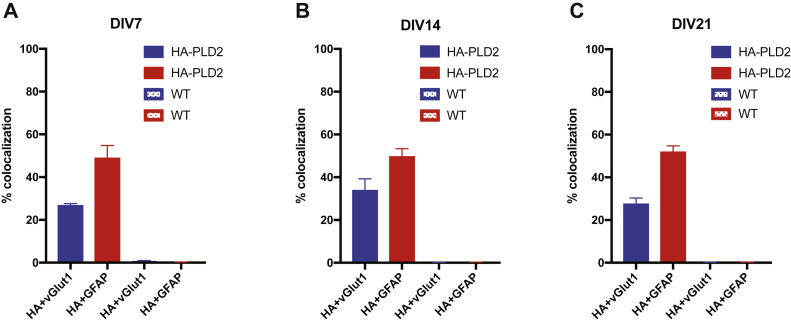
Quantification of HA-PLD2 and WT cortical neuron immunofluorescence. The percent colocalization was calculated for HA + vGlut1 and HA + GFAP in myc-PLD1 and WT cortical neurons at DIV7 (A), DIV14 (B), and DIV21 (C). Data is representative of three experiments and error bars represent SEM. A: HA-PLD2 n = 5, WT n = 3; B, HA-PLD2 n = 5, WT n = 3; C, HA-PLD2 n = 6, WT n = 2. GFAP, glial fibrillary acidic protein; vGlut1, vesicular glutamate transporter 1.

PLD2 expression in neurons and glial cells was also examined using HA-PLD2 mice. In contrast to PLD1, PLD2 expression is much higher in glial cells from the earliest timepoint and remains higher in glial cells through development into maturity. PLD2 expression is consistently lower in neurons than glia. Similar to PLD1, at DIV21, PLD2 staining colocalizes with vGlut1 in a punctate pattern, suggesting that PLD2 is also localized at the synapse at this time (Figs. 4A and 5A: HA + vGlut1 26.66 ± 0.95, HA + GFAP 48.86 ± 5.92; Fig. 5B: HA + vGlut1 33.79 ± 5.45, HA + GFAP 49.53±3.84; Fig. 5C: HA + vGlut1 27.43 ± 2.87, HA + GFAP 51.83 ± 2.91). As a control, the same staining protocol was performed in WT cortical cultures and there was no HA staining observed within these cells, suggesting that our HA antibody is also specifically recognizing PLD2 in these immunofluorescence experiments (Figs. 4B and 5A HA + vGlut1 0.74 ± 0.21, HA + GFAP 0.19 ± 0.08; Fig. 5B HA + vGlut1 0.08 ± 0.05, HA + GFAP 0.24 ± 0.07; Fig. 5C HA + vGlut1 0.12 ± 0.09, HA + GFAP 0.13 ± 0.12). Taken together, these experiments are the first to demonstrate that while both PLD1 and PLD2 are present in neuronal and glial cells, PLD2 expression is higher in glial cells and PLD1 expression is higher in neuronal cells. Notably, at DIV21, both PLD1 and PLD2 are enriched at synapses. This evidence for synaptic localization of PLD isoforms contributes to the hypothesis that PLDs are involved in regulating neurotransmission.

### PLD1 and PLD2 expression in mouse brain slices

**Fig. 6:**
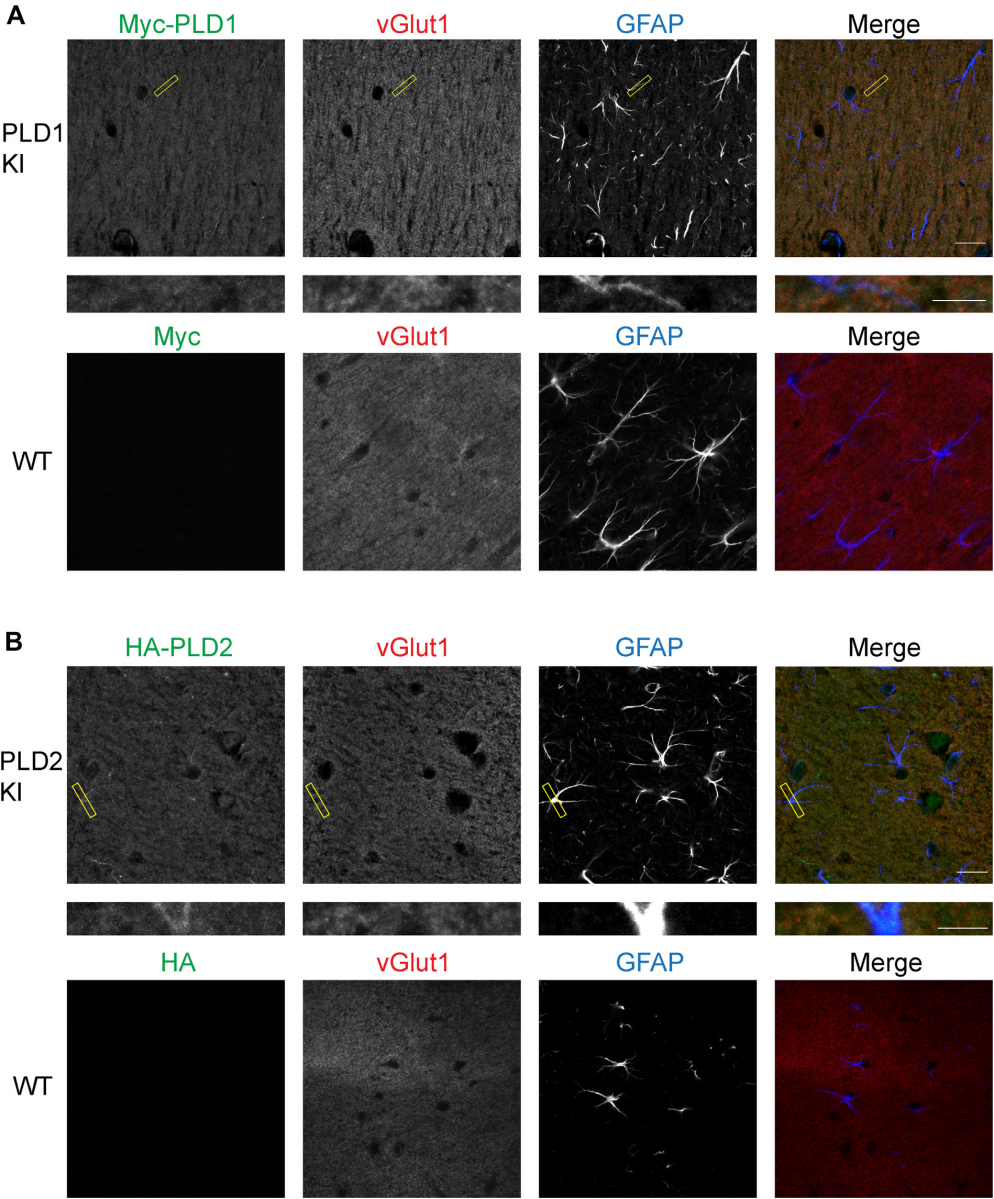
In brain slices, PLD1 and PLD2 are both expressed in neurons, with PLD2 expression higher in glial processes. A: P26 myc-PLD1 brains were fixed, sectioned, and stained with myc, vGlut1, and GFAP antibodies. No myc staining is observed in WT mouse (P25) cortical brain slices. B: P32 HA-PLD2 brains were fixed, sectioned, and stained with HA, vGlut1, and GFAP antibodies. No HA staining is observed in WT mouse (P25) cortical brain slices. Scale bars represent 20 μm and 5 μm (cropped region). Images are representative of three separate experiments. GFAP, glial fibrillary acidic protein; vGlut1, vesicular glutamate transporter 1.

**Fig. 7:**
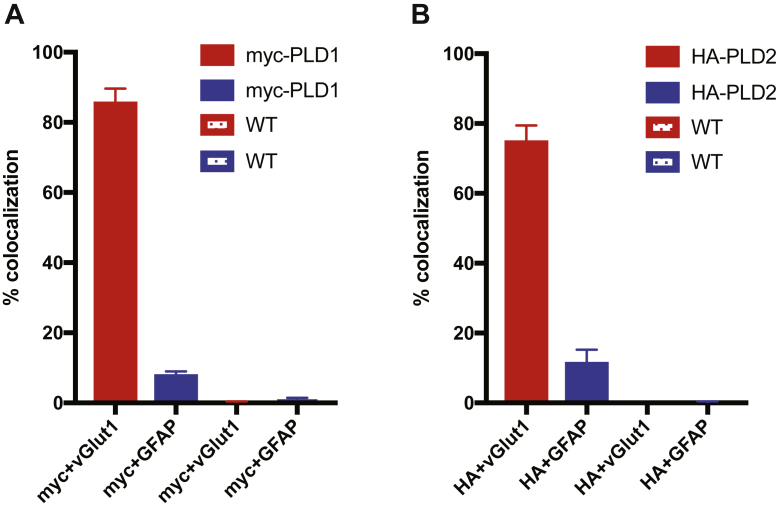
Quantification of myc-PLD1, HA-PLD2, and WT cortical brain slices. Percent colocalization of myc + vGlut1 and myc + GFAP was calculated for myc-PLD1 and WT cortical brain slices (A) and HA-PLD2 and WT cortical brain slices (B). Data is representative of two experiments (two mice) and error bars represent SEM. A: myc-PLD1 n = 3, WT n = 3; B, HA-PLD2 n = 3, WT n = 3. vGlut1, vesicular glutamate transporter 1; GFAP, glial fibrillary acidic protein.

Next, to complement our findings from the knockin culture staining, we examined PLD1 and PLD2 expression in knockin brain slices from adult mice. Whole brains from WT (P25), myc-PLD1 (P26), and HA-PLD2 (P32) mice were sectioned, fixed, and stained for myc/HA, vGlut1, and GFAP. In the myc-PLD1 mice, PLD1 expression seems to overlap with vGlut1 expression, further suggesting that PLD1 is localized synaptically at maturity. There was little myc staining that overlapped with GFAP staining, indicating that there is little PLD1 expressed in glia at this stage of development (Figs. 6A and 7A: myc + vGlut1 85.36 ± 4.22, myc + GFAP 7.68 ± 1.34). As with cultured neurons, when this staining protocol was performed in WT brain slices, very little myc staining was observed (Figs. 6A and 7A: myc + vGlut1 0.30 ± 0.17, myc + GFAP 1.02 ± 0.45). Staining for HA-PLD2 in mouse brain slices with HA, vGlut1, and GFAP showed that although diffuse, PLD2 expression also overlapped with vGlut1 staining, again suggesting PLD2 localization is synaptic. Via HA staining, PLD2 expression is also observed in glial processes (Fig. 6B and 7B: HA + vGlut1 74.7 0 ± 4.76, HA + GFAP 11.26 ± 4.00). No significant HA staining was observed in WT brain slices (Fig. 6B and 7B: HA + vGlut1 0.01 ± 0.00, HA + GFAP 0.29 ± 0.15). These findings complement our PLD2 staining in culture, suggesting that PLD2 is expressed in glial cells and neurons at maturity. Taken together, we observe that both PLD1 and PLD2 are located synaptically in mature brain slices, with PLD2 expression being predominant in glial cells. These findings complement our previous data and further demonstrate that PLD1 and PLD2 are enriched at synapses, supporting a potential role in neurotransmission.

### PLD1 and PLD2 expression in mouse retinal slices

**Fig. 8:**
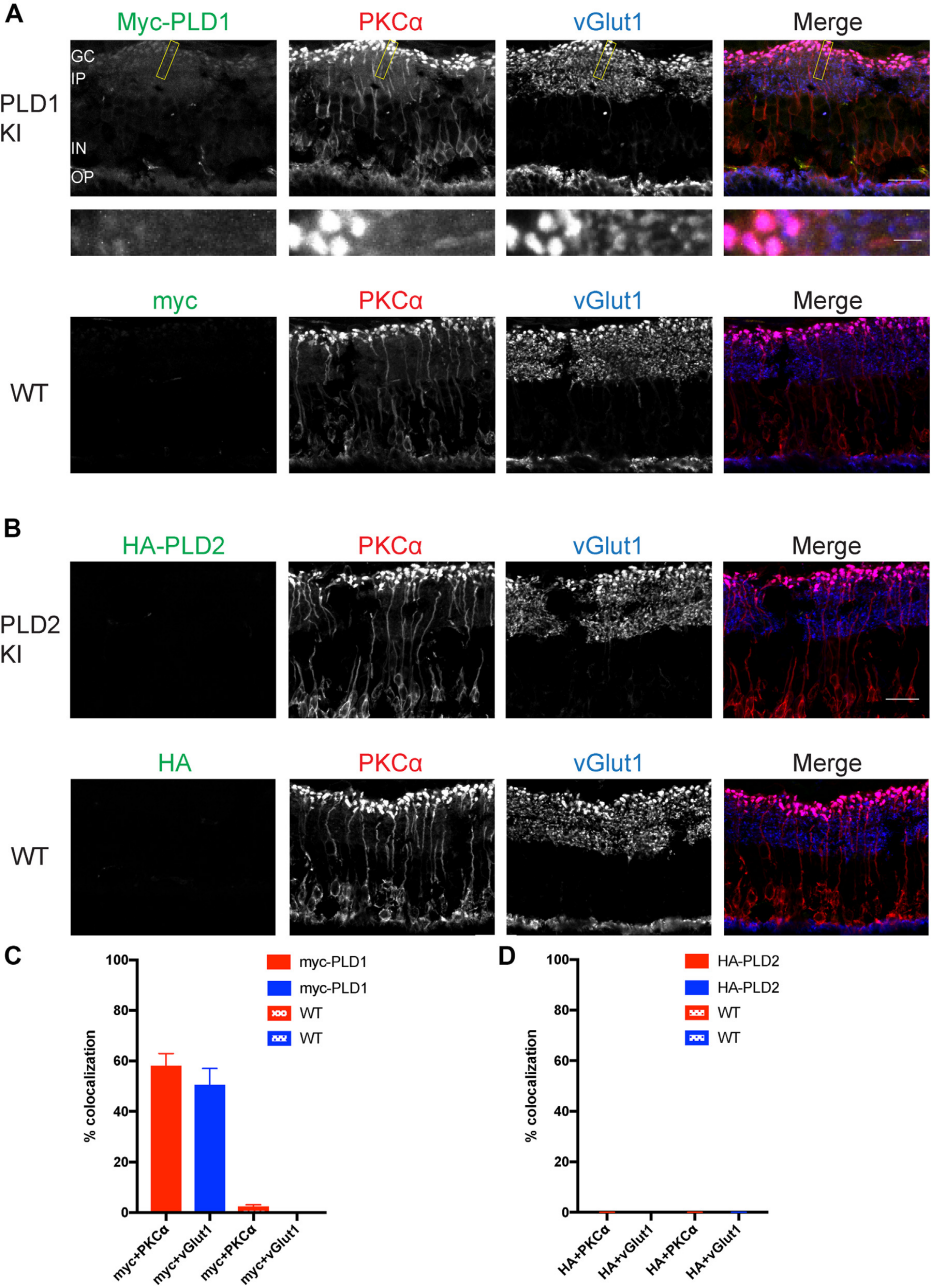
PLD1 exclusively is expressed in mouse retinal slices. A: Retinal slices from P55 myc-PLD1 mice were stained with a myc antibody to visualize PLD1 localization, a PKCα antibody to label bipolar and ganglion cells, and a vGlut1 antibody as a marker of presynaptic boutons. No myc staining is observed in P67 WT retinal slices. B: Retinal slices from P59 HA-PLD2 mice were also stained with HA, PKCα, and vGlut1 antibodies. No HA staining is observed in P67 WT retinal slices. Scale bars represent 20 μm and 5 μm (cropped region). Percent colocalization of myc + PKCα and myc + vGlut1 was calculated for myc-PLD1 and WT retinal slices (C) and of HA + PKCα and HA + vGlut1 for HA-PLD2 and WT retinal slices (D). Data is representative of three experiments and error bars represent SEM. C: myc-PLD1 n = 6, WT n = 5; D, HA-PLD2 n = 5, WT n = 5. Images are representative of three separate experiments. GC, ganglion cell layer; IP, inner plexiform layer; IN, inner nuclear layer; OP, outer plexiform layer; vGlut1, vesicular glutamate transporter 1.

The data above demonstrates that while PLD1 expression is predominant in neurons, both isoforms are expressed around neuronal synapses. This, together with previous data implicating PLD1 in acetylcholine release (31), leads to a strong hypothesis that PLDs, particularly PLD1, may be involved in modulating vesicle dynamics during neurotransmission. We reasoned that a role for a PLD may be particularly important in neurons where there is a high demand for synaptic vesicle cycling such as in sensory neurons found in the retina. Photoreceptors and bipolar cells of the retina have ribbon synapses, specialized synapses at which several hundreds to thousands of synaptic vesicles are released per second to transmit the graded synaptic output of visual information (32). Therefore, we stained retinal slices obtained from WT (P67), myc-PLD1 (P55), and HA-PLD2 (P59) mice with myc/HA, vGlut1, and PKCα antibodies. PKCα labels bipolar cells and ganglion cells, while, as mentioned above, vGlut1 serves as a marker of presynaptic, glutamatergic synapses. Interestingly, when we stained our knockin mouse retinal slices for PLDs, PLD1 appears to be the main PLD isoform expressed in the retina, as we observed minimal PLD2 expression in these sections (Fig. 8A–D, Fig. 8D: HA + PKCα 0.06 ± 0.03, HA + vGlut1 0.02 ± 0.00). Myc staining visualizing PLD1 expression diffusely colocalizes with PKCα expression, with some puncta that colocalize with vGlut1. This pattern suggests that PLD1 is localized diffusely to the synaptic plexiform layers (Fig. 8A, C: myc + PKCα 57.87 ± 5.12, myc + vGlut1 50.29 ± 6.74). Undoubtedly, more work is required to identify the exact localization of PLD1 in these layers. Staining of WT mouse retinal slices with the same staining protocols did not detect any staining with myc or HA antibodies (Fig. 8C: myc + PKCα 2.12 ± 0.97, myc + vGlut1 0.03 ± 0.01; Fig. 8D: HA + PKCα 0.06 ± 0.03, HA + vGlut1 0.04 ± 0.03). Taken together, this data suggests that PLD1 is the sole PLD isoform (with phospholipase activity) expressed in the mouse retina and is localized within the synaptic plexiform layers. This further strengthens the notion that PLD1 is involved in neurotransmitter release.

### Electroretinograms with WT and PLD1 KO mice

**Fig. 9:**
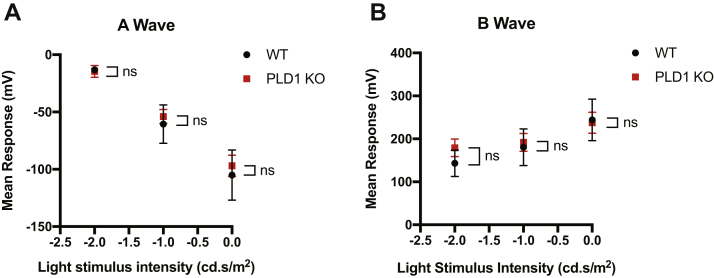
ERG recordings from WT and PLD1 KO mice reveal no significant differences in the A or B wave. WT and PLD1 KO mice (all P58) were dark adapted and ERG recordings were measured in response to a range of light intensities. The average a-wave amplitude (A) and b-wave amplitude (B) from responses are plotted by each light stimulus. Data analyzed with unpaired t-tests. A: −2.0 cd .s/m^2^*P* = 0.7953, −1.0 cd .s/m^2^*P* = 0.7471, 0.0 cd .s/m^2^*P* = 0.7676; B: −2.0 cd .s/m^2^*P* = 0.3876, −1.0 cd .s/m^2^*P* = 0.8327, 0.0 cd .s/m^2^*P* = 0.9168. n = 3 mice. Error bars represent SEM. ERG, electroretinogram.

With PLD1 being the sole PLD isoform with PLD enzymatic activity that is expressed in the mouse retina, we were curious about a functional role for PLD1 in the retina. We hypothesized that this finding could suggest a role for PLD1 in sensory neurotransmission at ribbon synapses in the retina. To test the functional implications of PLD1 expression in the retina, we used ERGs to assay the electrical activity of the cells in the retina in response to a range of light stimuli. For dark-adapted (scotopic) ERGs, mice were exposed to flashes of light, which induces rod activation. This allows for the functional examination of photoreceptors and the downstream retinal cells. The output of this measure is a characteristic waveform in which the a-wave confers the initial response of the photoreceptors to the stimulus, and the b-wave is a measure of the downstream bipolar cells’ response to photoreceptor activation (33). Upon analysis of the a- and b-wave, one can observe gross conclusions about the reactivity and connectivity of the cells in the retinal layers. Because PLD1 was the sole PLD isoform expressed in the retina, we performed ERGs on WT and PLD1 KO mice (all P58). Mice were dark adapted and ERG recordings were measured in response to light intensities ranging from 0.01 to 10 cd .s/m^2^. Surprisingly, no significant differences were observed between WT and PLD1 KO mice in analysis of the a- or b-wave at any of the stimuli used (Fig. 9A, B). These experiments suggest that PLD1-mediated PtdOH production is not required for neurotransmission in the retinal layers.

## Discussion

In an effort to provide a clear understanding of PLD expression and localization in the mammalian brain, we generated the first endogenously expressed, epitope-tagged PLD1 and PLD2 knockin mouse lines. Our studies demonstrate that PLD1 and PLD2 are both localized synaptically in neurons in vitro, using cultured neurons, and in vivo, using brain slices. PLD1 expression is predominant in neurons, while PLD2 expression is robust in glial cells. In addition to tissue from the central nervous system, we also examined retinal slices from our knockin mice. These data indicate that PLD1 alone is expressed abundantly in the synaptic plexiform layer. However, analysis of cellular connectivity in the retina via ERG recordings revealed no differences between WT and PLD1 KO mice, suggesting PLD1 is not required for efficient neurotransmission within these cells. Altogether, these studies permit a more definitive understanding of the expression of PLD isoforms in neuronal and glial cells. Additionally, the generation of the myc-PLD1 and HA-PLD2 knockin mouse lines will be beneficial to a wide range of studies regarding PLDs, from their antiapoptotic role in cancer (34, 35), to their potential as a therapeutic target for Alzheimer’s disease (11, 36, 37).

The results in this report will impact a range of other topics regarding functional roles of PLDs. Although PLD-mediated production of PtdOH is implicated in exocytosis, functional studies of PLD1 in neurons have also revealed a role for PLD1 in dendritic branching. Studies in rat hippocampal cultures concluded that overexpression of PLD1 led to a decrease in the complexity of dendritic arborization, while inhibition of PLD1 caused increased dendritic branching (25). Further, it was recently found that PLD1 interacts with PKD1 in neurons and positively regulates dendritic spine morphogenesis (38). Our data that PLD1 is expressed in neurons throughout development, into maturity, and is expressed at the synapse supports these findings. Myc-PLD1 cortical cultures display PLD1 expression robustly throughout dendrites. In contrast to these functional studies, the literature lacks concrete evidence about PLD1’s potential role in synaptic vesicle cycling. Its localization at synapses identified in our studies contributes to the hypothesis that PLD1 modulates synaptic vesicle exocytosis, also supported by the 2001 study that found the injection of catalytically inactive PLD1 led to a decrease in acetylcholine release (31). While this is still an exciting finding, it is important to recall that conceptually, the injection of dominant-negative proteins into neurons can have a wide range of unexpected effects (39, 40). The results from this study (31) also suggest that PLD1 affects the number of available vesicle release sites and not necessarily the release of vesicles at the remaining sites. Further studies, however, will be required to reach a definitive conclusion about the role of PLD1 in synaptic vesicle recycling. For example, future studies should investigate endocytic and exocytic rates of PLD KO neurons.

Our data suggests that PLD2 expression is robust in glial processes in brain slices and in culture through development and into maturity. The expression of a PLD in glial cells leads to interesting hypotheses about the production of PtdOH with regards to the well established regulation of neurotransmission by astrocytes (41, 42, 43). Astrocyte processes protrude into neuronal synapses and participate in innumerable functions that support the health of neurons and the efficiency of neurotransmission. Given their intimate proximity, astrocytes produce and traffic a wide variety of metabolites to neurons at the synapse (44, 45, 46) one of which could be PtdOH. For example, studies have suggested that exogenous addition of PtdOH to neuronal cultures can affect dendritic branching and other neuronal characteristics (24). Given our data, we hypothesize that PLD2 is likely responsible for PtdOH production in glia, and that PtdOH is trafficked from astrocytes to neurons at the synapse where it could function to regulate synaptic vesicle exocytosis or participate in other signaling roles. Therefore, PtdOH pools at synapses could have additional sources from astrocytes, as demonstrated previously (47).

While we did not intend to study the internal and subcellular localization of PLDs, we are able to make some general observations. Interestingly, PLD1 is highly expressed in the neuronal nucleus at DIV7 and 14, which is consistent with some previous investigations into PLD1 subcellular localization (48, 49). In contrast, PLD2 is obviously absent from the glial nucleus at all timepoints studied. Some studies report PLD1 and PLD2 to be localized within the Golgi and functions in Golgi vesicular transport (50, 51) and also report both PLD isoforms to be membrane localized (15, 51, 52). In our current findings, both PLD1 and PLD2 appear to be expressed within the synaptic region of neurons at DIV21.

Our data provides evidence that PLD1 is the sole PLD isoform with phospholipase activity that is expressed in the retina. Interestingly, Thakur *et al.*(53) found that PLD is light activated in photoreceptors of fruit flies. Supporting a role for PLD in the regulation of vesicular transport activated PLD coordinates endocytosis at photoreceptor membranes and therefore the recycling of rhodopsin to the cell surface. Overall, PLD regulates membrane homeostasis to maintain the size and rhodopsin composition of the membrane of these photoreceptors . We emphasize that further analysis is needed to determine the exact localization of PLD1 within the retinal layers.

ERGs studies, used to probe the potential functional role of PLD1 in the retina, found no significant differences between WT and PLD1 KO mice upon analysis of the a- and b-wave. While many studies have reported no major visual or behavioral defects in PLD KO mice (11, 54), more minor findings have recently been published (2, 22). Here, our finding suggests that PLD1 is not required for efficient neurotransmission within the retina, although additional studies are required to confirm this hypothesis. This could, in part, be explained by the fact that there are other sources of PtdOH in the retina that compensate for the loss of PLD1-mediated PtdOH production. The ribeye protein, the main component of the synaptic ribbon, possesses lyso-PA-acyltransferase activity that generates PtdOH from lyso-PtdOH (55). Because our ERG analysis is a general investigation into neural connectivity specifically in the retina, to directly address the function of PLDs in neurons, future studies should explore excitatory postsynaptic current recordings in PLD KO mice.

Overall, our localization studies with the novel epitope-tagged PLD1 and PLD2 knockin mice permit a more definitive understanding of the expression of PLD1 and PLD2 in the mammalian brain and retina. As such, it will benefit not only the field of lipid metabolism but neurobiology as well. Importantly, the data in this report will provide crucial information in assessing the functional role of PLDs in neurons and glia. Outside of a direct role in synaptic vesicle recycling, there is also potential for other signaling roles for PLD at the synapse. Furthermore, these knockin mice will be indispensable for future work investigating the cellular and subcellular localization of PLDs in a variety of tissues.

## Data availability

All data are contained within this article.

## Supplemental data

This article contains supplemental data.

## Conflict of interest

The authors declare that they have no conflicts of interest with the contents of this article.
