# Supplementary figures and images for: Differential expression patterns of phospholipase D isoforms 1 and 2 in the mammalian brain and retina
Source: J Lipid Res. 2022 Jun 25;63(8):100247. doi: 10.1016/j.jlr.2022.100247 (PMC9305353; doi:10.1016/j.jlr.2022.100247)

### PLD Activity

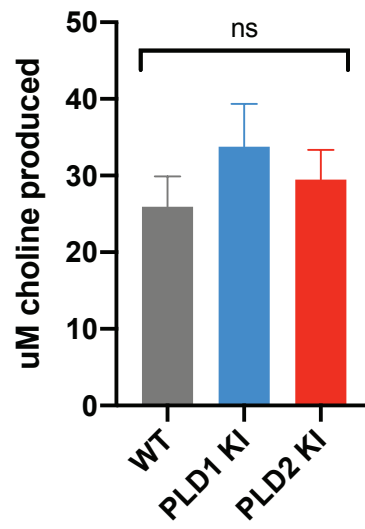

Supplement: Supplementary Figure 1 [file mmc1.pdf]
